# Missing data in multi-omics integration: Recent advances through artificial intelligence

**DOI:** 10.3389/frai.2023.1098308

**Published:** 2023-02-09

**Authors:** Javier E. Flores, Daniel M. Claborne, Zachary D. Weller, Bobbie-Jo M. Webb-Robertson, Katrina M. Waters, Lisa M. Bramer

**Affiliations:** ^1^Pacific Northwest National Laboratory, Biological Sciences Division, Earth and Biological Sciences Directorate, Richland, WA, United States; ^2^Pacific Northwest National Laboratory, Artificial Intelligence and Data Analytics Division, National Security Directorate, Richland, WA, United States

**Keywords:** data integration, missing data, multi-omics, multi-view, artificial intelligence, machine learning, neural networks, Bayesian

## Abstract

Biological systems function through complex interactions between various ‘omics (biomolecules), and a more complete understanding of these systems is only possible through an integrated, multi-omic perspective. This has presented the need for the development of integration approaches that are able to capture the complex, often non-linear, interactions that define these biological systems and are adapted to the challenges of combining the heterogenous data across ‘omic views. A principal challenge to multi-omic integration is missing data because all biomolecules are not measured in all samples. Due to either cost, instrument sensitivity, or other experimental factors, data for a biological sample may be missing for one or more ‘omic techologies. Recent methodological developments in artificial intelligence and statistical learning have greatly facilitated the analyses of multi-omics data, however many of these techniques assume access to completely observed data. A subset of these methods incorporate mechanisms for handling partially observed samples, and these methods are the focus of this review. We describe recently developed approaches, noting their primary use cases and highlighting each method's approach to handling missing data. We additionally provide an overview of the more traditional missing data workflows and their limitations; and we discuss potential avenues for further developments as well as how the missing data issue and its current solutions may generalize beyond the multi-omics context.

## Introduction

In recent years, advances in technology and decreased costs have resulted in an increase in the availability of high-throughput biological instrumentation to researchers. As a result, in the biological sciences, data are being generated at an unprecedented rate. The generation of multiple ‘omics data types on the same set of samples is more commonly referred to as multi-omics. Motivated by the hope for a more holistic understanding of a biological system, researchers now regularly conduct multi-omics research generating multiple ‘omics data types (e.g., transcriptomics, proteomics, etc.) for the same study for numerous research areas such as biomedicine, soil science, microbiology, and plant science. Each distinct ‘omics data type provides unique information about a specific level of the biological system's process. For example, transcriptomics technologies can measure ribonucleic acid (RNA) molecules in an organism and gives researchers a picture of which genes are being expressed (Liang, 2013). Proteomics instrumentation can measure the relative abundance of proteins that are produced from gene expression and transcript translation (Graves and Haystead, 2002).

Although the generation of multi-omics data is becoming commonplace, many challenges remain in the analysis of multi-omics data. The analyses of individual ‘omics datasets continues to be challenging for computational scientists due to the size and complexity of the individual datasets and continued introduction of new technologies and instrumentation. However, data integration is one of the primary barriers to effective multi-omics research and reaching the desired holistic view of the biological system being studied (Misra et al., 2019). This has resulted in a large body of literature and reviews dedicated to multi-omics data integration in the last 10 years. Several of these review articles have focused specifically on the availability of software tools and discussions of preprocessing considerations (e.g., Arakawa and Tomita, 2013; Misra et al., 2019), or on providing perspective on challenges in multi-omics integration (e.g., Gomez-Cabrero et al., 2014). Some reviews focus on differentiating general data-driven integration approaches from a mathematical perspective (e.g., Ritchie et al., 2015; Bersanelli et al., 2016; Noor et al., 2019), focus on a particular type of method, such as dimension reduction (Meng et al., 2016), or compare data-driven models against biologically-informed models for a particular model type, such as network models (Wanichthanarak et al., 2015) with little attention on particular ‘omics data types. On the other hand, many of these articles focus on integration for specific ‘omics data types, such as single cell ‘omics (Ma et al., 2020) or specific application areas, such as plant biology (Rajasundaram and Selbig, 2016), microbial communities (Fondi and Liò, 2015; Franzosa et al., 2015), cancer (Buescher and Driggers, 2016), and others (e.g., Subramanian et al., 2020).

Hinderances to effective integration methods include, but are not limited to, the heterogeneous nature of data and distributional properties across multi-omics datasets, the typically large difference in the number of measured biomolecules compared to number of samples or replicates, and the complex and often noisy nature of biological data. Machine learning (ML) and artificial intelligence (AI) have shown promise in overcoming some of these hindrances (e.g., Mirza et al., 2019; Zhu, 2020) and have gained popularity in recent years. Consequently, further review articles have been written placing emphasis on ML and AI for multi-omics data integration. Included in these are articles discussing examples of method application (e.g., Holzinger et al., 2019; Li et al., 2021) and challenges in applying AI and ML techniques to multi-omics data (e.g., Kang et al., 2021; Termine et al., 2021). Many are focused on specific application domains such as metabolic engineering (Helmy et al., 2020), precision medicine (Hamamoto et al., 2019), amongst others (e.g., Mann et al., 2021; Lin et al., 2022; Zhou et al., 2022) and a majority focused on cancer research (e.g., Wang and Gu, 2016; Biswas and Chakrabarti, 2020; Nicora et al., 2020; Cai et al., 2022). Finally, a handful of reviews are dedicated to discussion of subsets of general AI and ML approaches to multi-omics data integration (e.g., Li et al., 2016; Huang et al., 2017; Kim and Tagkopoulos, 2018; Picard et al., 2021; Reel et al., 2021; Lee and Kim, 2022) with some attention given to limitations of methods, such as interpretability of models.

One limitation of AI and ML models is that a majority of methods require complete data with no missing observations, requiring a user to discard data for any biomolecule with a missing value, remove samples with any missing values, or impute missing values, either before input into the model or implicitly done by the model's software implementation, or some combination of these options. Discarding data is typically not the preferable option for multi-omics data analysis, due to data types with high amounts of missing data or complex mechanisms behind missing observations. For example, the human proteome project reports that an estimated ~20% of genes yield protein products that are not detected by mass spectrometry, an analytic platform for protein quantification (Paik et al., 2012; Baker et al., 2017). Furthermore, additional complexities including protein isolation and solubilization, sequence ambiguity, and a lack of standards in statistical thresholds and algorithms result in inconsistent detection of large proportions of protein products that are detected, thus limiting the reproducibility of data collection and analysis (Goh and Wong, 2017). In metabolomics, limited coverage of the known metabolome increases the risk of overlooking the metabolomic response of interest in targeted metabolomic analyses. Non-targeted analyses offer the potential to determine novel biomarkers, but these analyses are inherently biased as an analyst's selection of specific instrumental parameters (e.g., stationary phase and ionization mode) induces increased instrumental sensitivity toward some substances and reductions toward others (Ribbenstedt et al., 2018).

Given the prevalence of missing data across different ‘omics, the approach that ML and AI models use in handling missing observations is of great importance when considering multi-omics integration methods. Despite the importance of considering methods for handling missing observations, this topic is either not addressed or is only briefly mentioned in existing multi-omics data integration review articles. In this review, we describe recently developed integration approaches with specific attention on each method's approach to handling missing data. We additionally provide an overview of the more traditional missing data workflows and their limitations. Finally, we discuss potential avenues for further method development as well as how the missing data issue and its current solutions may generalize beyond the multi-omics context.

## Background

### Missing data and imputation

Missing data are common in ‘omics data and can arise due to a variety of reasons, such as poor tissue quality, insufficient sample volume, measurement system limitations, budget restrictions, or subject dropout. Taking proteomics as a specific example, peptide identification is done within the context of a mass spectrometry run, and the peptides observed and identified vary from run to run. Peptides may be missing from one or more samples due to a variety of underlying mechanisms (Daly et al., 2008; Karpievitch et al., 2009), which can result in non-trivial amounts of potential observations being missing. It is not uncommon to have 20–50% of the possible peptide values that are not quantified (Webb-Robertson et al., 2015; Brenes et al., 2019). It has been shown that missing values in large-scale ‘omics data can hinder downstream analyses (Ouyang et al., 2004; Jörnsten et al., 2005). As a result, the handling of missing data is a regular challenge in the integration and analysis of multi-omics data. For data integration, missing data is especially challenging because the set of observations with missing data and the proportion of missingness can vary among the different ‘omics datasets.

There are three classifications that are typically used to describe the mechanisms generating missing values (Rubin, 1976), and it is common practice to assume that one of these classifications characterize the missing data of a given sample. Missing data may be classified as missing not at random (MNAR), missing at random (MAR), or missing completely at random (MCAR). MCAR is a special case of MAR and occurs when the missingness does not depend on other variables and can be considered purely stochastic (Wei et al., 2018). An assumption of MCAR implies an equal probability of missingness for all observations and that this probability does not depend on observable or unobservable features. Missing values that are MAR are those with missingness that is stochastically independent of the measurand and depends only on other observed variables (van den Boogart and Tolosana-Delgado, 2013). When an assumption of MCAR or MAR is used, the missingness is said to be ignorable and methods designed to handle missing values are applied to both cases (Gelman and Hill, 2006; Fang et al., 2018; Wei et al., 2018). MNAR broadly describes when an assumption of MAR is violated. It can occur when missing values depend on other unobserved variables or when the missingness depends on the value itself. For example, data that are missing due to the limit of detection or quantification (LOD/LOQ) and not through the influence of other measured variables in the data are considered MNAR. In ‘omics studies, data are more commonly MNAR or MAR, considering that instruments are known to have certain detection thresholds (MNAR) and that biochemical dependencies exist between different biomolecules (MAR).

Complete case and available case methods are among the simplest approaches for dealing with missing data. Within a multi-omics context, a complete case analysis considers only the set of subjects with completely observed features across all measured ‘omics. This approach, while convenient, results in a decrease in sample size, throws away useful data, is only valid under the MCAR assumption and can otherwise lead to biased estimates (Fang et al., 2018; Hawinkel et al., 2020). An available case analysis uses different, complete subsets of the data to answer various research questions (Gelman and Hill, 2006). While more robust than a complete case analysis since less data are dropped, an available case analysis is still only valid under the MCAR assumption and generates conclusions based on different parts of the data, potentially leading to biased or conflicting inference.

Imputation approaches offer an alternative route for handling missing data that circumvents the complete and available case analysis issues of utilizing only subsets of the full dataset. These approaches impute, or fill in, missing values based on values of the observed data. Mean imputation, for example, replaces each missing value by the mean of the observed data in its corresponding feature. While this simple approach preserves feature means and grants access to the use of more data, it does not preserve relationships among variables and may lead to an underestimate of variability (Gelman and Hill, 2006). Other simple approaches include zero-value or LOD imputation, both of which are often applied when data are missing at random or censored. However, like mean imputation, imputed values from these approaches may distort feature distributions and lead to biased parameter and variability estimates (Lubin et al., 2004; Succop et al., 2004; Webb-Robertson et al., 2015; Lazar et al., 2016; Bramer et al., 2020). These tradeoffs are characteristic of most of these more naïve imputation methods– one avoids the pitfalls of complete case analyses at the expense of introducing potential biases in the imputed data.

The broader research community has thus introduced more sophisticated imputation approaches that mitigate the drawbacks of more naïve methods (Enders, 2010). Examples include linear regression models that have been utilized to reflect the relationship between observed variables and those with missing entries to inform imputation (Hair et al., 2010; van Buuren and Groothuis-Oudshoorn, 2011). Matching approaches, like hot deck or cold deck imputation, can be used to find observations that are like those with missing values. Missing values may then be imputed with the values from the most similar observations. Along these lines, the K-nearest-neighbors (KNN) imputation approach, which may be viewed as a hybrid of the regression and matching approach (Gelman and Hill, 2006), identifies an observation's k closest neighbors, and uses the mean of these neighbors to fill in the missing value. KNN imputation has been shown to work well for numerical imputation on single datasets (Jadhav et al., 2019), but the algorithm is sensitive to the choice of the number of nearest neighbors (k) and is limited in that it does not utilize the potential structure across the entire set of observations. Other common imputation approaches include random forest imputation (Pantanowitz and Marwala, 2009) and expectation maximization (Dempster et al., 1977). Discussions of approaches well-suited for single ‘omics imputation are provided by Webb-Robertson et al. (2015) and Bramer et al. (2020).

Each of the previously described imputation methods have generally been developed within the context of a single dataset, however multi-omics data are uniquely characterized by several separate, yet related, datasets. This aspect of multi-omics has therefore motivated the development of several newer approaches that leverage information across different ‘omics datasets to inform imputations. One example, TDImpute (Zhou et al., 2020), provides a transfer-learning approach for the imputation of gene expression data from DNA methylation data. In this method, the weights of a fully connected neural network trained on the publicly available Cancer Genome Atlas (TCGA) dataset are fine-tuned through additional training on a target dataset. Then, predictions generated by this fine-tuned model are used to impute missing values in the target dataset. Peacock et al. (2022) generalize this approach, using the TCGA dataset to train networks for the imputation of DNA methylation data from gene expression data, micro RNA data from gene expression data, and gene expression data from methylation data. Howey et al. (2021) develop a different solution, proposing a modified nearest-neighbors algorithm based on Bayesian multi-omics networks. For single-cell multi-omics imputation, Eltager et al. (2022) introduce yet another variation on the KNN method by imputing single-cell transposase accessibility chromatin (scATAC-seq) data based on nearest neighbors identified with corresponding single-cell transcriptomics (scRNA-seq) data. Trans-Omics Block Missing Data Imputation (TOBMI; Dong et al., 2018) proposes a similar method, but imputed values are generated based on a weighted average of neighbors, with closer neighbors having greater weights. Last, Ni et al. (2022) introduce scLRTD, an imputation approach based on approximating a third-order tensor representation of the multi-omics dataset with a low-rank approximation. For a more thorough review of recent imputation approaches as they relate to multi-omic analyses, we refer the reader to Song et al., 2020.

Given the ubiquity of partially observed cases in multi-omics datasets—and that most integrative multi-omics methods are not inherently compatible with partially observed data—imputation methods are often incorporated as a data pre-processing step within the multi-omics analytic pipeline, or alternatively, partially observed samples are dropped and a complete case analysis is performed. The smaller subset of integrative approaches that are compatible with, and have mechanisms for handling, missing data are the focus of the present review. Prior to our discussion of these missing-data-robust methods, we first describe the more general class of multi-omics integrative methods and highlight a few representative approaches that are not necessarily attuned to the missing data problem.

### Multi-omics integration

A wide variety of techniques are available that leverage the information contained in the relationships between ‘omics types. Development of these methods have been driven by different applications (i.e., pathway analysis vs. drug response prediction) and the various challenges specific to multi-omics data analysis such as inter-omic data heterogeneity (e.g., imbalanced number of features, different distributions/missingness patterns) and high-dimensionality. Despite the multitude of approaches, there are enough commonalities to motivate a rough framework for their categorization into broader methodological groups.

Integration techniques generally assume measurements from the same samples on several markers, or features, from several ‘omics-data types, which are more generally referred to as data “views”. These markers may include gene, protein, and metabolite expression levels that measure the presences and relative abundance of these biomolecules within a sample. The techniques that perform integration can be roughly organized by how they combine each view. A sensible partition is of early, middle, and late integrative methods, alternatively denoted as concatenation, transformation-based, or model-based integration, respectively (Ritchie et al., 2015). Early integration is primarily characterized by an initial concatenation of the features across all measured ‘omics, followed by application of methods appropriate for analyzing the resulting high-dimensional dataset. Middle integration applies some transformation to represent a complex combination of the datasets before applying downstream analyses. Late integration analyzes each dataset separately and introduces a model or algorithm that combines the outputs of each individual analysis. Reviews of ‘omics integration methods (Nicora et al., 2020; Picard et al., 2021) attempt to categorize the various approaches by more specific characteristics or techniques, including feature selection, feature extraction or latent representations, kernel learning, matrix factorization, graph/network representations, and deep learning. Picard et al. (2021) further separate the early-middle-late categorization into early, mixed, late, intermediate, and hierarchical. These categorizations are not mutually exclusive, and a given method may be difficult to classify, but we briefly describe some examples that illustrate various distinctions and, for each example, mention possible weaknesses to the missing data problem.

Xie et al. (2019) use deep learning to perform survival analysis, which involves feature selection by removing observations with high missingness, *early concatenation* of raw features to form the input to their model, and implicit feature extraction using deep neural networks, as the value at each hidden layer possibly forms a useful representation. This approach can be distinguished from “middle” or mixed integration by noting that the model *immediately* considers all features. For contrast, another deep learning-based method (Xu et al., 2019) trains separate networks that see only the data from a particular view and then fuses the hidden representations at some intermediate layer. Both these methods require complete input, and initial imputation is performed in both cases to accommodate. Given the tendency of neural networks to overfit to the training data, it is possible that any bias in the imputation of the training data—which is likely due to the common violation of MAR in ‘omics data—will be erroneously exploited by the network.

The method introduced by Koh et al. (2019) discovers interactions between biomarkers by representing them as nodes in a graph, with edges representing some notion of similarity or interaction between them. They perform a form of feature selection by extracting sub-graphs that are useful for predicting phenotype. This is an example of middle integration due to the transformation of the raw input into an intermediate graph representation before analysis. Another graph method, netDx (Pai et al., 2019), represents patients as nodes and constructs a separate graph among the patients for each biomarker or group of biomarkers. These graphs are scored according to their ability to correctly classify patients and those passing a certain score threshold are selected for downstream analysis. The separate construction of graphs for disjoint sets of features suggests categorization as a middle integration method. Similarly, kernel-based methods often construct kernel representations of each dataset separately as pairwise relationships between samples or biomarkers. Middle integration can be performed through combining the kernels as in Mariette and Villa-Vialaneix (2018) and Gönen and Alpaydin (2011). These methods rely on an imputation pre-processing step, which could possibly negatively affect the downstream analyses, as the relationships between nodes/samples/biomarkers may be biased toward similarities in imputation. If an entire view is missing for one or more samples and must be imputed, any level of similarity specific to that view may be lost.

There is a large class of algorithms that Picard et al. (2021) categorize as intermediate methods. These generally form view-specific representations and assume a common latent space among views (Yang and Michailidis, 2015; Chalise and Fridley, 2017) or maximize some measure among the view-specific representations (Tenenhaus and Tenenhaus, 2011; Meng et al., 2014; Tenenhaus et al., 2017; Singh et al., 2019). Picard et al. (2021) state that these methods often require robust preprocessing to reduce heterogeneity between datasets, suggesting careful selection of imputation techniques that do not introduce view-specific bias. Though not unique to these methods, some (Tenenhaus and Tenenhaus, 2011; Singh et al., 2019) employ the technique of user-specified connections (or lack of) between variables. A similar approach can be seen in the Bayesian network method of Howey et al. (2021). This approach introduces prior knowledge about the relationships between biomarkers, allowing for the construction of a hierarchical organization of relationships between layers of the biological system.

Late integration examples include the method introduced by Sun et al. (2019) that processes each ‘omic type separately through a deep neural network (DNN) and then performs score fusion on the predictions. The introduced framework is such that the DNN for each view could be replaced by any one of several algorithms. Another example, MOLI (Sharifi-Noghabi et al., 2019), similarly processes each view separately through a DNN and concatenates the multiple latent representations before sending them through a classifier network.

The vast majority of these integration approaches require completely observed data for their implementation. When faced with missing data, these methods thus require a complete case analysis or the implementation of separate imputation steps beforehand. Missing samples in multi-omics data are rarely MCAR, and thus inference based on a complete case analysis is likely to be biased. A separate imputation procedure—particularly those that are more computationally sophisticated—is also suboptimal from an efficiency standpoint. More ideal would be those approaches that explicitly address the missing data problem within their methodology, being able to utilize partially observed cases and incorporate mechanisms that remove the need for imputative pre-processing steps. Recent examples of such approaches are the focus of this review and are described in the next section.

## Integrative approaches for partially observed multi-omics

Broadly speaking, integrative approaches that explicitly address the missing data problem do so in one of two ways. Missing data are either jointly imputed within the specified modeling framework or algorithm, or a flexible optimization routine is implemented such that the missing components of partially observed samples are masked while their observed components are still able to contribute to parameter estimation. We refer to the former as joint-imputation approaches, and the latter as optimization-masking approaches. In the following subsections, we describe recently developed joint-imputation and optimization-masking approaches, grouping each under integrative approach classifications consistent with those defined by Ritchie et al. (2015). For reader convenience, Table 1 provides a more concise summary of each described method, where the integration and missing data approach categorizations, compatible ‘omics data types, primary use case, application data sample characteristics, and software availability information are listed.

**Table 1:** Integrative approaches for partially observed multi-omics data.

**Integration approach**	**Missing data approach**	**Method**	**Compatible omics platforms**	**Primary use case**	**Application dataset**	**Software availability**
Early Integration	Joint-Imputation	FBM	Gene expression and methylation data	Feature selection and Prediction of clinical outcomes (regression)	Children asthma data: 460 samples, >2,000 features across DNA methylation and RNA-Seq gene expression data	R scripts available here: https://github.com/CHPGenetics/FBM
		iMODA	Any, provided compatibility with their proposed causal structure	Identification of phenotypic associations across multi-omics (regression)	SPIROMICS and COPDGene data (includes proteomic and genomic data): 2,974 samples, 600,000+ features	Custom software downloadable here: https://dlin.web.unc.edu/software/iMODA/
	Optimization-Masking	TiMEG	Genotype, gene expression, and methylation data	Identification of disease-associated biomarkers (regression)	Dataset on Tuberous Sclerosis Complex patients: 8,036 gene expression data on 34 samples, 481,470 methylation features on 29 samples, and 1,298,477 genotype features for 45 samples	R scripts available here: https://github.com/sarmistha123/TiMEG
Middle Integration	Joint-Imputation	MOFA/MOFA+	Any	Dimension reduction (clustering)	(MOFA) CLL data (includes somatic mutation status, transcriptome profiling, and DNA methylation data): 200 samples, 9,000+ features (MOFA) Mouse single-cell data (includes DNA methylation and transcriptome data): 87 samples, 9,000+ features (MOFA+) single-cell datasets (includes DNA methylation, gene expression, and chromatin accessibility data): sample sizes range from 2,000 to 16,152 samples, 9,000+ features	Python package (mofapy2) and R package (MOFA2) available. Installation instructions found here: https://biofam.github.io/MOFA2/installation.html
		BIDIFAC+	Any	Bi-dimensional integration and matrix factorization for dimension reduction	TCGA dataset: 6,973 samples, 1,000 mRNA expression features, 732 miRNA expression features, 1,000 methylation features, and 198 proteomic features	R scripts available here: https://github.com/lockEF/bidifac
	Optimization-Masking	COMBI	Any	Dimension reduction (clustering)	HMP2 data (includes proteome and microbiome data): 132 samples, feature count not specified Zhang data (includes microbiome and immunological data): sample size or feature count not specified Gavin data (includes microbiome and human and microbial proteome data): 55 samples, number of features not specified	R package (combi). Installation instructions found here: https://github.com/CenterForStatistics-UGent/combi
		MVAE	Any	Generative model training for multi-modal inference	Four image datasets (MINST, FashionMNIST, MultiMNIST, CelebA): number of sample and features not specified	None
		DeepMF	Any	Cancer subtype detection (clustering)	Medulloblastoma dataset (includes mRNA data): 34 samples, number of features not specified Leukemia data set (includes mRNA data): 38 samples, number of features not specified TCGA BRCA dataset (includes mRNA, miRNA, and protein data): 150 samples, number of features not specified SRBCT dataset (includes mRNA data): 63 samples, number of features not specified.	Python scripts available here: https://github.com/paprikachan/DeepMF
		MvNE	Any	Dimension reduction (clustering)	TCGA datasets (includes gene expression, miRNA expression, and DNA methylation data): sample sizes ranging from 200 to 600; over 25,000 features across three omics	None
		DeepIMV	Any	Dimension reduction for classification or prediction	TCGA dataset (includes mRNA expression, DNA methylation, miRNA expression, and protein data): 7,295 samples, number of features not specified CCLE dataset (includes DNA copy number, DNA methylation, mRNA expression, miRNA expression, protein, and metabolite data): 504 samples, number of features not specified	Python scripts available here: https://github.com/chl8856/DeepIMV
		JACA	Continuous omics data; sample labels are additionally required	Identification of associations between views (e.g., omics) that are pertinent to their classifications	A TCGA dataset: 282 samples, 1,572 RNAseq features and 375 miRNA features	R package (JACA). Installation instructions found here: https://github.com/Pennisetum/JACA
		SUMO	Any, but benchmarked using continuous genomic data types	Molecular subtype detection (clustering)	The Cancer Genome Atlas (TCGA) datasets: sample sizes ranging from 200 to 600; over 25,000 features across several omics (methylation, gene expression, and miRNA expression)	Command-line tool (requires Python 3.6+). Installation instructions found here: https://github.com/ratan-lab/sumo
Late Integration	Optimization-Masking	NEMO	Any	Cancer subtype detection (clustering)	Digits dataset: 500 samples, < 1,000 features across several data views TCGA datasets: sample sizes ranging from 2,00 to 600; over 25,000 features across several omics (mRNA expression, DNA methylation, and miRNA expression)	R scripts available here: https://github.com/Shamir-Lab/NEMO
		MSNE	Any	Cancer subtype detection (clustering)	Digits dataset: 2,000 samples, < 1,000 features across several data views TCGA datasets: sample sizes ranging from 200 to 600; over 25,000 features across several omics (mRNA expression, copy number variation, DNA methylation, and miRNA expression)	Python scripts available here: https://github.com/GaoLabXDU/MSNE
		MONET	Any	Cancer subtype detection (clustering)	Digits dataset: 400–2,000 samples, < 1,000 features across several data views TCGA datasets: sample sizes ranging from 200 to 600; over 25,000 features across several omics (mRNA expression, DNA methylation, and miRNA expression)	Python scripts available here: https://github.com/Shamir-Lab/MONET. R code in development

### Early integration

The methods described in this section are those that are concatenation-based. Therefore, their implementation assumes that the separate ‘omics datasets collected for a set of samples are concatenated into a single, high-dimensional matrix.

#### Joint-imputation

The full Bayesian model with missingness (FBM; Fang et al., 2018) extends the iBAG model (Wang et al., 2012) that was introduced for modeling associations between gene expression, methylation, and a clinical response. The iBAG model is a two-layer hierarchical Bayesian model whose first layer (i.e., the “mechanistic model”) assesses the effect of methylation on gene expression and whose second layer (i.e., the “clinical model”) models the relationship between the clinical outcome, gene expression, and other clinical factors (e.g., age and gender). The iBAG approach assumes completely observed data, and to remedy this, the FBM approach introduces a third, imputation layer that models missing gene expression and methylation data through multivariate normal distributions. The FBM model uses a Gibbs sampling algorithm for simultaneous model parameter estimation and missing value imputation, where missing values are imputed as draws from estimated posterior distributions. Simulation studies performed in the original publication indicate increases in the root mean squared error of prediction with increases in missing data percentage. This effect was observed to be most prominent when both gene expression and methylation data were characterized by high percentages of missingness (i.e., 30%). However, the observed decreases in predictive performance were observed to be mitigated as the sample size was increased. Also worth noting is that the FBM model assumes gene-gene and methylation-methylation independence and imputed values reflect this assumption accordingly. However the authors note that models incorporating gene-gene and methylation-methylation correlations were additionally considered, but these models were associated with a higher computational cost without indicating substantive gains in performance relative to models based on the independence assumption.

Emphasizing analyses more consistent with a causal framework, iMODA (Lin et al., 2020) proposes a hierarchical regression framework for quantifying (i) the effects of genotypes on quantitative ‘omics, (ii) the effects of quantitative ‘omics on response phenotypes of interest, and (iii) the direct and indirect effects of genotypes on these response phenotypes. The indirect effect of genotype on phenotype refers to the effect mediated by ‘omic expression, whereas the direct effect refers to the genotype effect with indirect effects removed. The proposed framework is aligned with the known causal hierarchy across these biomolecules, i.e., genotype affects ‘omic expression which influences phenotype. Missing ‘omics data are accommodated through an expectation-maximization (EM) approach (Dempster et al., 1977) for parameter estimation. Implementation of the EM algorithm involves iterating between an expectation step, where missing values are imputed based on the parameter estimates of a previous iteration, and a maximization step, where parameter estimates are updated based on the imputed data, until parameter estimates converge. The power of the iMODA framework to detect the effects of interest has been reported to vary according to the proportion of data that are missing. The authors considered cases where 50% and 30% of data were missing, finding that power decreases with increased missingness.

#### Optimization-masking

Introduced by Das and Mukhopadhyay (2021), TiMEG (Tool for integrating Methylation, gene Expression and Genotype) is a supervised learning approach for the identification of disease-associated biomarkers in case-control multi-omics studies. TiMEG utilizes a hierarchical logistic regression modeling framework to relate a binary response (e.g., disease status) to a linear combination of different ‘omic predictors. Different layers of TiMEG's hierarchical framework are specified to model known biological relationships between these different ‘omics. For example, gene expression is known to be affected by changes to the DNA sequence. To account for this, TiMEG includes a layer that regresses gene expression level on genotype and methylation, factors that both reflect DNA sequence alterations. The TiMEG model is fitted through maximum likelihood estimation, and to accommodate any partially observed data, approximations are used when constructing the model likelihood. More specifically, TiMEG's robustness to missing data is primarily enabled by an approximation that involves representing the logistic component of the likelihood by the cumulative distribution function of a normally distributed random variable. This allows for cancellations within the joint-likelihood that effectively mask the missing portions of sample observations, without dropping such cases entirely. As with other missing data approaches, simulation studies indicate that the power of the TiMEG approach varies with the degree of missingness. As the percentage of missing data increases, the power decreases. However, across changes in missing data percentages, the type 1 error rate is controlled at the nominal rate.

### Middle integration

Contrasting the integrative approaches of the previous subsection, the following methods are middle, or transformation-based, integration approaches. In general, each of these approaches involves the mapping of the multi-omics data to some lower-dimensional space containing latent representations of different combinations of input features spanning multiple omics.

#### Joint-imputation

Multi-Omics Factor Analysis (MOFA; Argelaguet et al., 2018) and its more recent improvement, MOFA+ (Argelaguet et al., 2020) are unsupervised integrative approaches that may be understood as generalizations of principal components analysis (PCA) to multi-omics data. These approaches factorize each ‘omics data matrix into products of two component matrices, with one component matrix shared across all ‘omics matrix factorizations. The shared component matrix generates a low-dimensional representation of the data that accounts for the variability across each of the individual ‘omics datasets and may be used for downstream analyses (e.g., clustering). MOFA's factorization model is formulated within a probabilistic Bayesian framework such that regularization for feature sparsity may be implemented through the specification of priors on the unobserved model variables (i.e., component matrices). Parameters are updated according to an EM-like algorithm, whose implementation enables the incorporation of partially observed data points. The MOFA+ algorithm was introduced to address MOFA's scaling issues by implementing a stochastic variational inference framework for model estimation, substantially speeding up computations. As a joint-imputation approach, MOFA/MOFA+ was compared to other imputation strategies including imputation by feature-wise mean, SoftImpute (Mazumder et al., 2010) and a k-nearest neighbor approach (Troyanskaya et al., 2001). Comparing these approaches in a simulated setting, it was found that imputation by MOFA resulted in a lower mean squared error of prediction (i.e., imputation) relative to compared methods across varying percentages of missingness. The missing percentages considered ranged between ~10–~80%, and all methods demonstrated increases to mean squared error of prediction with increased missing percentage.

Unique from most other approaches described in this review, BIDIFAC+ (Lock et al., 2022) is a matrix factorization approach for bi-dimensionally linked matrices. Traditional multi-omics considers the integration of several different ‘omics for the same set of samples, whereas the integration considered by BIDIFAC+ involves combining different ‘omics and different sample sets. BIDIFAC+ builds upon BIDIFAC (Park and Lock, 2019) and SLIDE (Gaynanova and Li, 2019), two extensions of the JIVE method (Lock et al., 2013) that yields matrix factorizations that convey a decomposition of data variability into variability shared across multiple ‘omics and variability primarily attributable to single ‘omics platforms. The proposed BIDIFAC+ model is amenable to a probabilistic representation and is compatible with a modified EM algorithm approach to jointly impute missing data during model parameter estimation. Simulation studies assessing the performance of BIDIFAC across different types of missingness (entry, column, and block) and signal strength indicate that for entry-level missingness, imputation error is smallest when for stronger signals. For column-missingness, where entries for an entire ‘omic are missing across all samples, imputation error is smallest for stronger joint-signal strength. A similar trend is observed for block-level missingness, which is the type of missingness where several omics (columns) are missing across a set of samples (rows).

#### Optimization-masking

Building upon the matrix factorization model introduced by MOFA (Argelaguet et al., 2018), Compositional Omics Model-Based Integration (COMBI; Hawinkel et al., 2020) introduces model extensions better suited for modeling compositional and sequence count data. Sequence count data describe the type of data common to transcriptomics and other ‘omics, and these ‘omics data are also compositional given that they indicate sample composition as opposed to direct molecular concentrations. To better conform to the compositional nature of these data, COMBI modifies the MOFA regression formulation of the matrix factorization process by specifying a centered log-ratio transform (clr) link function. The inverse of the clr function is the softmax function, whose output is a vector with elements that sum to one (i.e., a composition). COMBI utilizes quasi-likelihood estimation (Wedderburn, 1974) to fit its model, enabling one to specify only a mean and variance as opposed to an entire parametric distribution for model estimation. Modeling only the mean and variance through quasi-likelihood estimation is advantageous considering that sequence count data are often characterized by high variability and inflated zero-counts, aspects which are often poorly modeled by fully specified parametric distributions. The quasi-likelihood estimation approach is also robust to missing data values. Provided that the data are missing completely at random (MCAR) or missing at random (MAR), the quasi-likelihood estimation procedure yields unbiased parameter estimates based on all (complete or partial) observations. Missing values of a given ‘omic are simply excluded from the estimating equations.

Wu and Goodman (2018) propose a multimodal variational autoencoder (MVAE), an unsupervised approach for integrative multi-view (i.e., multi-omic) analyses. MVAE's use of variational autoencoders (Kingma and Welling, 2014) casts the encoders and decoders of a traditional autoencoder within a stochastic framework, allowing one to leverage certain probabilistic assumptions and properties in specifying the loss function for model training. One key assumption made by this approach is that data views are independent from one another, given a shared latent representation. This assumption, and a few other approximations, motivate a model architecture and loss function described by the authors as a product-of-experts (PoE) that efficiently combines the variational parameters of ‘omic-specific encodings when mapping to a lower-dimensional, shared latent space. This PoE model architecture is robust to partially observed samples, combining only the ‘omic-specific encodings of each partial sample's observed ‘omics during model training.

DeepMF (Chen et al., 2019) casts traditional matrix factorization within a deep learning framework to map concatenated multi-omics data to a lower-dimensional latent space. This is achieved by specifying a fully connected network architecture that takes as input one-hot encoded representations of each ‘omics feature, and outputs predicted values of the original multi-omic data matrix. Between these input and output layers are several (at least two) hidden layers that enforce a lower-dimensional representation of the input one-hot encodings. To train this network, a variational L2-norm is used such that missing ‘omics entries may be dropped during backpropagation. The trained network yields three matrices of potential use to the analyst. The first is the predicted data matrix, whose entries may be used to impute any missing data values in the original data matrix. The second extracted matrix is the “feature-latent” matrix, which is obtained by taking the trained weights of the first hidden layer. The columns of this “feature-latent” matrix define the relative weights of each original feature in generating the corresponding latent factor (column) of the matrix. The third extracted matrix, the “sample-latent” matrix, defines a similar mapping, but here, each row defines the relative weights of each original sample in defining the corresponding latent factor. This matrix is obtained by taking the trained weights of the final hidden layer that precedes the output layer. A natural use of the feature-latent or sample-latent matrices are for clustering analyses to identify feature or patient subgroups. In evaluating the robustness of DeepMF to varying levels of missingness (10, 50, and 70%), Chen et al. (2019) found that DeepMF was consistently able to identify sample and feature subgroups with perfect accuracy. However, it should be noted that these evaluations were completed in a simulated data setting in which data were simulated as missing completely at random (MCAR) and ground truth feature subgroups were generated with simple discrimination boundaries.

A similarly motivated approach, the Multi-view Neighborhood Embedding (MvNE) method introduced by Mitra et al. (2020), integrates and maps multi-omics data to a lower-dimensional subspace to facilitate cluster analyses. The proposed model first estimates a unified probability distribution that describes the likelihood of neighboring points across the full-dimensional space of the original multi-omics data. This distribution is “unified” in the sense that it represents an aggregation of individual, ‘omic-specific neighbor distributions. Aggregation of these normal distributions is achieved through the process of statistical conflation (Hill, 2011), which involves taking the normalized product of the aggregated probability density functions. To map the data to a lower-dimensional subspace, embeddings are generated by a stacked autoencoder, and these embeddings are optimized by iteratively minimizing the Kullback-Leibler divergence (Kullback and Leibler, 1951) between the unified probability distribution of the full-dimensional data and the neighborhood distribution based on the lower-dimensional embeddings. Samples with partially observed ‘omics data are incorporated during optimization through adjustments to the conflation approach used to generate the unified neighborhood distribution.

DeepIMV (Lee and van der Schaar, 2021) incorporates some of the principles utilized by Wu and Goodman (2018) to develop a deep variational information bottleneck approach that maps integrated, multi-omics data into a lower-dimensional subspace of task-relevant embeddings. These task-relevant embeddings are those attuned for either regression or classification purposes, improving upon a similar approach (Zhang et al., 2019) whose embeddings are generated in an unsupervised fashion. DeepIMV's latent mappings are trained according to the informational bottleneck principle (Tishby et al., 2000; Alemi et al., 2016; Achille and Soatto, 2017), which seeks to define task-relevant data representations that respect the balance between parsimony and predictive power. The DeepIMV approach is characterized by a model architecture that may be partitioned into four components: view-specific encoders that map the data of individual ‘omics to a common latent space; a product-of-experts (PoE) network module that maps the marginal latent representations of the view-specific encoders to a joint latent representation within the same common latent space; a multi-view predictor that generates task-specific predictions based on the joint encodings of the PoE module; and view-specific predictors that generate predictions based on the view-specific (marginal) encodings. Use of the PoE module in the DeepIMV architecture allows one to ignore missing ‘omics for a partially observed sample without dropping the sample entirely during model training. Though the marginal encodings or ‘omic-specific predictions associated with these missing ‘omics may not be generated for these partially observed samples, the PoE module may still flexibly use only the marginal encodings of these sample's observed ‘omics to generate joint latent encodings. These joint encodings may then be used by DeepIMV's multi-view predictor to generate response predictions. The robustness of DeepIMV to different rates of missingness was assessed by the authors using several benchmark datasets provided by the Cancer Genome Atlas (TCGA) and the Cancer Cell Line Encyclopedia (CCLE). Using the area under the receiver-operator curve (AUROC) as a performance metric, the authors found that the performance of DeepIMV diminished with increased missingness but performed as well as or better than the compared approaches.

Joint association and classification analysis (JACA; Zhang and Gaynanova, 2021) is a semi-supervised approach that enables quantification of inter-omic associations with respect to known classifications or data subtypes. JACA establishes a connection between linear discriminant analysis (LDA) and canonical correlation analysis (CCA) to motivate the construction of a loss function that combines and balances between the objectives of each approach. Balance between these objectives is regulated by a tuning parameter, α. When α = 0 the JACA loss reduces to that of CCA or SUMCOR multiset CCA (Kettenring, 1971) with sparsity regularization, depending on whether three or more ‘omics are under consideration. On the other hand, when α = 1, JACA reduces to sparse LDA with additional orthogonality constraints. JACA's loss function utilizes a co-regularization framework, where a regularization term is shared across omic-specific loss functions. The JACA loss may be viewed as an aggregation of individual objective functions—specific to individual ‘omics—that are linked and jointly optimized by their shared regularization. Similar co-regularization approaches for semi-supervised multi-omic or (more generally) multi-view learning have been previously introduced (Sindhwani et al., 2005; Brefeld et al., 2006; Sun and Shawe-Taylor, 2010). Missing data may be accommodated by JACA provided that partially observed samples have either a response label and at least one observed ‘omic, or at least two observed ‘omics. For either case, the loss function is constructed as it is under the complete-case setting, except that each loss component may have a different subset of contributing samples. Partially observed samples with a response label and at least one observed ‘omic are used to train the omic-specific classifiers (i.e. LDA component) whereas partially observed samples of the other variety are used to penalize inter-omic classification disagreement (i.e., CCA component).

Last, SUMO (Sienkiewicz et al., 2022) is a non-negative matrix factorization approach that enables the integration of continuous data from multiple data types to infer subtypes in multi-omics datasets. Implementation of SUMO involves the construction of ‘omic-specific similarity matrices, where entries of the similarity matrices are computed as radial basis functions of the Euclidean distances between sample pairs. Each similarity matrix is then factorized into two-non-negative matrices— one representative of the basic components and the other containing mixture coefficients indicative of cluster membership. This factorization is done jointly across all similarity matrices such that the matrix of mixture components is shared across all similarity matrices, similar in spirit to the shared factorization of MOFA and MOFA+. The iterative solver used to determine this joint factorization is specified such that missing similarity entries are masked. Intuitively, this is achieved by viewing each similarity matrix as a connected graph and missing entries as edgeless nodes within this graph. The authors compared SUMO to a different integrative approach, NEMO (Rappoport and Shamir, 2019), in terms of their robustness to varying levels of missing samples. These comparisons indicated a slight outperformance by SUMO, except for in the cases of extreme missingness (80% and greater). As well, the range of adjusted rand index values (the performance metric used) was typically observed to be narrower for SUMO, suggesting less variability in expected performance.

### Late integration

Here we describe integrative approaches for partially observed multi-omics that may be categorized as late-integrative, or model-based, since each involve some *post-hoc* combination of several ‘omic-specific analyses. Of the recent approaches surveyed by this review, only optimization-masking approaches were identified.

#### Optimization-masking

Neighborhood based Multi-Omics clustering (NEMO; Rappoport and Shamir, 2019) is an algorithm that generates clusters based on individual data view similarity matrices. NEMO operates in three phases, where in the first phase similarity matrices and relative similarity matrices are built separately for each data view (i.e., ‘omics data type) under consideration. The relative similarity matrix of a data view is a function of its similarity matrix, and its entries capture the similarity between two points relative to the nearest neighbors of each point. In the second phase of NEMO these relative similarity matrices are averaged across views, and in NEMO's final phase, spectral clustering is performed on the averaged relative similarity matrix. NEMO can handle partially observed samples, provided that these samples are observed in at least one view common to other observed samples. For these cases, the relative similarity matrix entries for a given point pair are simply averaged across the data view matrices where both points (i.e., samples) have observed values of the data view. A more recent approach, Multiple Similarity Network Embedding (MSNE; Xu et al., 2021) modifies NEMO's original framework to overcome limitations in the patterns of missing data that can be accommodated. MSNE incorporates a random walk strategy for integrating across view-specific similarity matrices, leading to a more flexible framework for handling missing data and removing the condition that partially observed samples may only be integrated if they are observed in at least one data view common to other observed samples.

Multi Omics clustering by Non-Exhaustive Types (MONET; Rappoport et al., 2020) is a clustering algorithm that defines “modules”, which are clusters formed based on subsets of different data views. Modules are detected by identifying heavy subgraphs common across the separate, edge-weighted graphs constructed for each view. This detection process is guided by an objective function defined by the sum of module weights, where the weight of a module is the sum of all edge weights between all sample pairs across all ‘omics in the module. This algorithm incorporates partially observed samples by representing their missing components as edgeless nodes within their corresponding edge-weighted graphs. Notably, there is no constraint on the number of detected modules, nor is there an upper bound on module size. As a result, maximizing the objective function for heavy subgraph detection is an NP-hard problem. The authors circumvent this issue by proposing a heuristic algorithm that identifies a local maximum and recommend using the best solution from repeated runs of the algorithm to better approximate the globally maximal solution.

## Discussion

Integrative approaches for the analysis of multi-omics data are rapidly evolving, and such advancements are largely driven by the power of AI and ML. In this work we have highlighted several of these more recent developments, focusing on those that directly embed, within their analytic frameworks, techniques that address the missing data issue that commonly arises in multi-omic data analysis. The reviewed approaches vary in their general applicability and use case but should be applied where appropriate to fully leverage the information across multi-omics datasets. However, in applying these methods, users should be aware of their limitations.

A limitation common to several approaches, for example, is the implicit requirement of relatively larger sample sizes for model estimation or fitting. We describe this requirement as “implicit” given that it is rarely, if ever, stated as a limitation, but instead implied based on the sample sizes of the datasets that these methods were successfully applied to. The datasets considered by these approaches contain hundreds, if not thousands, of observations, sample sizes that may be practically prohibitive due to cost, biospecimen availability, or both. For example, a study to identify biomarkers for Snyder Robinson syndrome, a rare neurological disorder, obtained data on 16 patients, only three of which presented with the disorder of interest (Abela et al., 2015). Other studies that involve rare diseases, the collection of difficult-to-access tissue, or smaller grants that do not allow for large consortium samples are characterized by similarly low sample sizes (Sirrs et al., 2015; Abela et al., 2017). These cases present the need for methods that generate reliable inference despite smaller sample sizes and avoid overfitting to the data. Alternatively, the development of methods for integrating multi-omics data generated by separately conducted studies should be prioritized. The present review describes one of these methods, BIDIFAC+, that enables bi-dimensional data integration such that data may be integrated both vertically (i.e., integration across omics) and horizontally (i.e., integration across sample cohorts). However, Lock et al. (2022) note that BIDIFAC+ assumes that shared structures are present across the integrated matrices, an assumption that may be violated in practice. As well, they noted that BIDIFAC+ took nearly 24 h to reach convergence when fitted to a combined TCGA dataset, and thus the method may not scale well with larger aggregations of datasets.

Though the shared structure assumption made by BIDIFAC+ may pose as problematic in certain practical instances, the ability to capture dependencies and correlations across ‘omics data types is important when considering that known biological relationships exist between these data. For joint-imputation approaches, which “predict” missing values, capturing data dependencies is particularly important as imputed values may be otherwise biased, affecting downstream results. Of the joint-imputation approaches discussed in this review, only the FBM and iMODA approaches describe ways in which the correlation structure is modeled or impacts results. Further investigations toward the effects of properly modeling these correlations are therefore warranted. On the other hand, though dependencies exist between different ‘omics data, important differences between ‘omics must also be accounted for in developed integrative approaches. Kok et al. (2018) notes, for example, that one may need around 19 samples per group to detect a fold change of 1.5 in microRNA data at 80% power and false discovery rate <10%. This is as opposed to other (larger) transcriptomics platforms that would require at least 35 samples per group to detect the same magnitude fold change at the same power and false discovery rate levels (Krassowski et al., 2020). This disparity in sample size requirements indicates differences in variability across different omics, implying inter-omic differences in signal strength as measured by signal-to-noise ratios (SNR). Thus, approaches that naïvely combine these data views may inadvertently overrepresent relationships particular to a high SNR ‘omics and underrepresent lower SNR ‘omics. Beyond variability and signal strength, heterogeneity across ‘omics may also be observed in terms of their missing data patterns or confounders, especially considering that different ‘omics are collected on different instrumentation that vary in their accuracy, precision, and detection ability.

Related to this point, many of the methods described here were motivated by or evaluated exclusively on sequencing-based omics platforms (e.g., transcriptomics, genomics, and epigenomics) and few considered the fuller breadth of omics platforms that includes metabolomics, proteomics, lipidomics, etc. Contrasting with the sequencing-based ‘omics used by many of the methods reviewed in this work, data of other ‘omics are typically generated by a mass spectrometer, an instrument whose use introduces several nuances to the data which may mean integration model assumptions reasonably made for sequencing-based data may not hold for mass spectrometry-based data. For example, batch effects are common and often much more pronounced in mass spectrometry-based data (Mertens, 2016; Phua et al., 2022; e.g., Han and Li, 2020), even when the same physical instrument is used and the same data type is being generated. Variations in sample handling, temperature fluctuation, imprecise timing, liquid chromatography/gas chromatography (LC/GC) column degradation and other factors result in systematic errors or biases of the measured abundances between the batches or over time. Further, mechanisms for missing data can be more complex in the case of mass spectrometry data, as described for proteomics data previously in this paper. For small molecule measurements, such as metabolomics, manual verification of metabolites following identification scoring is required for shotgun metabolomics, as scoring methods are much less mature than for other ‘omics data types (Stein and Scott, 1994; Samaraweera et al., 2018). This can result in increased misidentifications and missing values compared to ‘omics data with more established identification algorithms such as transcriptomics and genomics. Finally, additional sources of uncertainty may arise from processes such as protein quantification in bottom-up proteomics (Rifai et al., 2006; Plubell et al., 2022) as measurements are made at the peptide level and isoforms can be difficult to identify (Forshed et al., 2011; Webb-Robertson et al., 2014). In untargeted metabolomics and lipidomics false discovery rates are difficult to compute and largely not accurate (Stein, 1994; Jeong et al., 2011; Matsuda et al., 2013; Kim and Zhang, 2014). Additional exploration and evaluation of the appropriateness of the aforementioned integration methods is necessary given the differences in mass spectrometry-based data types compared to sequence-based omics data types.

While all these methods have been presented within the context of multi-omics integration, few explicitly require the use multi-omics data. The criteria of sets of features from distinct views across a shared set of samples is applicable to any domain where the combination of different data sources provides a more complete representation of the system under investigation. For example, many of the deep-learning architectures mentioned in this review have similarities to ones used in analysis of digital media that try to combine multiple modalities to improve predictive performance of video content (Carreira and Zisserman, 2017; Afouras et al., 2019). These methods also commonly investigate differences in “early”, “middle”, and “late” fusion and work has even been done to address the view-missing and view-heterogeneity problems (Nagrani et al., 2018; Wang et al., 2020). Insights from the ‘omics domain, where some of these issues are most salient, could transfer to other domains. The methodological choices inherent to the ‘omics domain are usually the ones that offer the ability to constrain or elucidate relationships between biomarkers. Some common choices are the use of principal-component-like representations that might identify important groups of biomarkers, and the injection of prior knowledge through techniques that constrain the relationships between features such as directed edges in graph representations, hard parameter constraints, or carefully chosen priors in Bayesian frameworks.

These biologically informed constraints are likely critical to develop successful approaches, as relying on over-parametrized models such as DNN's to learn non-spurious, inspectable relationships is not a reliable strategy (Lee and Kim, 2022). However, the strength of over-parametrized models in learning complex relationships cannot be ignored. The methodology discussed within this review spans the interpretability spectrum, from more interpretable approaches like iMODA (Lin et al., 2020) to more opaque methods such as DeepIMV (Lee and van der Schaar, 2021); and incorporating domain knowledge may be key toward bridging the interpretability gap apparent across these methods. For example, many of the middle integration methods discussed here are black-box in nature, as the specific relationships learned by each are often incommunicable. However, the groupings implicit within the latent factors generated by these models may be contextualized according to domain-specific knowledge (e.g., shared molecular pathways), thus increasing their interpretability and allowing one to infer underlying relationships. Identifying these groupings within latent factors is often a *post-hoc* process, however, and such domain-relevant groupings are not always identifiable within generated latent factors. If the generation of these latent factors were instead guided by domain knowledge, resulting groupings may be more likely to hold relevance and rely less on *post-hoc* analyses for contextualization within the scientific domain. Thus, recognizing the need for greater interpretability of the more opaque integration methods, efforts to combine biological-knowledge-injecting methods such as graph representations or other hard constraints on parameter representations with machine learning methods are ongoing (Noor et al., 2019; Lee and Kim, 2022), and evaluation and development of mechanisms for handling missing data within these frameworks will be necessary.

## Author contributions

JF, DC, ZW, and LB wrote the article. B-JW-R and KW revised the article. All authors contributed to the article and approved the submitted version.
